# Clinical Outcome of lateral mass screws for traumatic sub-axial facet dislocation

**DOI:** 10.12669/pjms.41.4.11894

**Published:** 2025-04

**Authors:** Sabir Khan Khattak, Sajid Hussain, Latif Khan, Ibtisam Haider, Amer Aziz

**Affiliations:** 1 Sabir Khan Khattak, Department of Orthopedic and Spine Surgery, Ghurki Trust and Teaching Hospital, Lahore, Pakistan; 2 Sajid Hussain, Department of Orthopedic and Spine Surgery, Ghurki Trust and Teaching Hospital, Lahore, Pakistan; 3 Latif khan, Department of Orthopedic and Spine Surgery, Ghurki Trust and Teaching Hospital, Lahore, Pakistan; 4 Ibtesam Haider, Department of Orthopedic and Spine Surgery, Ghurki Trust and Teaching Hospital, Lahore, Pakistan; 5 Amer Aziz, Department of Orthopedic and Spine Surgery, Ghurki Trust and Teaching Hospital, Lahore, Pakistan

**Keywords:** Cervical Spine, Facet Dislocation, Lateral Mass Screw, Neck Disability Index

## Abstract

**Objective:**

This study aimed to analyze the functional and neurological outcome of patients diagnosed with subaxial cervical spine bilateral facet dislocation managed by standard posterior midline approach and lateral mass screw fixation by Margerl technique

**Methods:**

We retrospectively evaluated 22 patients with traumatic cervical spine injuries who presented at the Orthopaedics and Spine Centre Ghurki Trust Teaching Hospital, Lahore from March 2020 to October 2023. Patients included in this study who has subaxial cervical spine bilateral facet dislocation managed by standard posterior midline approach and lateral mass screw fixation by Magerl technique. Functional outcomes was assessed by neck disability index and ASIA impairment scale at last follow up. Preoperative and post operative neurological status was evaluated with ASIA impairment scale. All the data were analyzed using SPSS version 23.

**Results:**

Total 22 patients participated in the study of which 72.73% were male and 27.27% female. Mean age was 39±17.02(13-70) years. Regarding level of dislocation, most common level was C5-6 which was involved in 12 patients, followed by C3-4 and C4-5 which was involved in five patients each. Pre-operatively 50% of the patients had intact neurology (ASIA E), followed by ASIA D which made up of the 27.27% patients, ASIA C 9.09%, ASIA B patients (4.55%), ASIA A 9.09% illustrating the severity of the neurological dysfunction. Postoperatively no neurological recovery was observed in ASIA A or ASIA B patients, while all ASIA C and D patients showed complete recovery. Mean Neck Disability Index (NDI) was 21±19.44.

**Conclusion:**

After a good reduction, lateral mass screws fixation are a safe and reliable approach for cervical fixation that not only stabilizes the cervical spine but also leads to a patient’s excellent functional recovery.

## INTRODUCTION

Traumatic injuries of the subaxial spine account for about 65% of all traumatic cervical spine injuries.^1^ Among them, facet dislocations and subluxations are the most severe form of injuries resulting in tetraplegia in up to 87% of the cases. Cervical facet dislocations are flexion-distraction injuries that mostly occur due to motor vehicle accidents. In a facet dislocation, the inferior facet of the superior vertebra is forced anteriorly relative to the superior facet of the inferior vertebra, which leads to ‘perched’ or locked facets. Unilateral facet dislocation results in 25% translation of one vertebra over another, while bilateral facet dislocation mostly results in 50% translation.^2^ Associated fractures include rare vertebral body fractures and relatively common posterior element injuries.

Bilateral facet dislocations are highly unstable injuries with associated neurological deficits and significant soft tissue injury. Urgent reduction, stabilization and decompression are needed for improved clinical outcomes.^3^ Closed reduction can be performed by applying Gardner-Well tongs. Initially, 2.5 to 5kg traction weight followed by 2-5kg for each level above dislocation, with incremental weights assessment of neurological status and screening with X-ray for reduction is mandatory, once closed reduction is achieved definitive surgical fixation can be done by either anterior cervical discectomy and fusion (ACDF) or posterior lateral mass screws (LMS) or anterior and posterior 360° fusion.^4^ Anterior cervical decompression and fusion are indicated in cases where a retropulsed disc compromises the canal, it allows restoration of cervical lordosis and reduces tension on the posterior ligamentous complex. Posterior cervical reduction and fusion have the advantage of direct visualisation and reduction of fracture/dislocation, decompression and a biomechanically stronger fixation. Despite advances in surgical techniques and methods of fixation, the optimal surgical approach remains controversial.^4^-^8^

Our part of the world which is underdeveloped and populous has a high incidence of cervical spine injuries mostly because of motor vehicle accidents. There are a few specialised centres where cervical spine trauma is being managed, so the local literature about this topic is scarce. We studied functional outcomes of bilateral cervical facet dislocation managed by standard posterior midline approach and lateral mass screws with mergels technique. We hope that our contribution will add further information to the already existing pool of knowledge and help in the advancement of surgical techniques and fixation methods in cervical spinal trauma.

## METHODS

This retrospective study was conducted at Orthopaedics and Spine Centre Ghurki Trust Teaching Hospital, Lahore between March 2020 and October 2023. Total 22 patients with traumatic cervical spine injuries confirmed on xray, CT scan with 3D reconstruction and MRI and fulfilled inclusion criteria were included in the study. Consecutive sampling technique was used and the sample size was based on available cases rather than a formal power calculation.

### Ethical approval:

The study was approved by the hospital ethical committee (Ref. No .2024/04/R-20, dated April 1, 2024).

### Inclusion & Exclusion Criteria:

The study’s patients had bilateral facet dislocations of the subaxial cervical spine, which were treated using the usual posterior midline technique with Magerl technique for lateral mass screw fixation. The age of the participants may vary from 13 to 70 years. Patients with bilateral facet dislocation with intact facets and fit for surgery of either age with no prolapsed anterior disc were included. While patients with fracture of cervical facets or pervious neck surgery, infection or tumor and not fit for surgery were excluded.

### Posterior approach with Magerl technique for lateral mass screw fixation:

Standard posterior midline incision was given, after dissection lateral mass was exposed then centre of lateral mass was identified, entry point was made at slightly medial and cranial to the mid point with the screw parallel to the adjacent facet and 20-30 degree of lateral angulation.^9^

### Rehabilitation:

The aim of rehabilitation is early recovery and out of bed mobilization, we started early out of bed mobilization and in bed physio to those with prior neurology loose. A cervical collar was adsvised to patients for six weeks to prevent uncontrolled neck movements.

### Followup time:

Six months followup after surgery was done. At the final follow-up, the neck disability index (NDI)^10^ and American Spinal Injury Association (ASIA) Impairment Scale^11^ was used to measure functional results. A ten-item questionnaire called the NDI is used to gauge a patient’s level of disability brought on by neck pain. Every item was given a score between 0 and 5, and the total percentage score was determined. No disability is represented by a score of 0-8%, mild disability by 10-28%, moderate disability by 30-48%, severe disability by 50-68%, and complete disability by greater than 68%. The ASIA Score was used to assess neurological state both before and after surgery.

**Fig:1:**
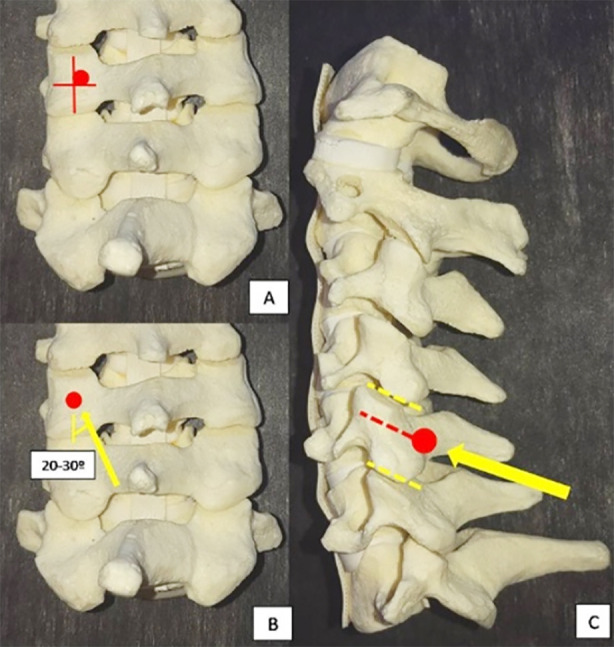
Magerl technique of LMS fixation.^9^

**Table-I:** Patient demographics and clinical characteristics.

Parameters	n	%
Total no. of patients	22	
Age (years)		39±17.02 (13-70)
*Gender*		
Male/Female	16/6	
Operation Level		
C3-C4	5	
C4-C5	5	
C5-6	12	
*ASIA Pre-op Neurology*		
Type A	2	9.09
Type B	1	4.55
Type C	2	9.09
Type D	6	27.27
Type E	11	50
*ASIA Post-op Neurology at six months*		
Type A	2	9.09
Type B	1	4.55
Type C	-	
Type D	-	
Type E	19	86.36
Neck Disability Index (NDI) (%)		21±19.44
No Disability	6	27.27
Mild Disability	13	59.09
Moderate Disability	1	4.55
Severe Disability	1	4.55
Complete Disability	1	4.55

### Statistical Analysis:

Data was analyzed using SPSS 23. Categorical variables, such as gender, level of injury, ASIA score pre-operatively and post-operatively were expressed in frequency and percentage, whereas continuous or quantitative variables such as patient’s age was expressed in mean±SD with range. Chi square test was applied and P-value of ≤0.05 was taken as significant.

## RESULTS

Out of the total 22 patients, 72.72% were male and 27.27% were female.The average age was 39^ί^17.02 years (13–70 years). In terms of dislocation level, the most prevalent level was C5-6, which affected 12 patients, followed by C3-4 and C4-5, each of which affected five patients. Pre-operatively, neurological assessments based on the ASIA score indicated that 11 patients (50%) were classified as Grade-E, six patients (27.27%) as Grade-D, two patients (9.09%) as Grade C, one patient (4.55%) as Grade-B, and two patients (9.09%) as Grade-A. Post-operatively, 19 patients (86.36%) demonstrated intact neurological function and were classified as Grade-E, reflecting significant improvement or conservation of neurological status. However, two patients (9.09%) remained at Grade-A, exhibiting complete neurological deficits, while one patient (4.55%) was classified as Grade-B, showing partial neurological impairment. Notably, the number of patients with intact neurological function (Grade-E) improved from 11 to 19 after surgical intervention, indicating neurological improvement in seven patients.

The Neck Disability Index (NDI) was used to evaluate functional outcomes, with a mean score of 21 ± 19.44%. Among the participants, six patients (27.27%) reported no disability, 13 patients (59.09%) experienced mild disability, and one patient each (4.55%) reported moderate, severe, and complete disability. These findings indicate that most patients achieved favorable functional recovery post-operatively, with the majority experiencing either no or mild disability.

**Fig.2:**
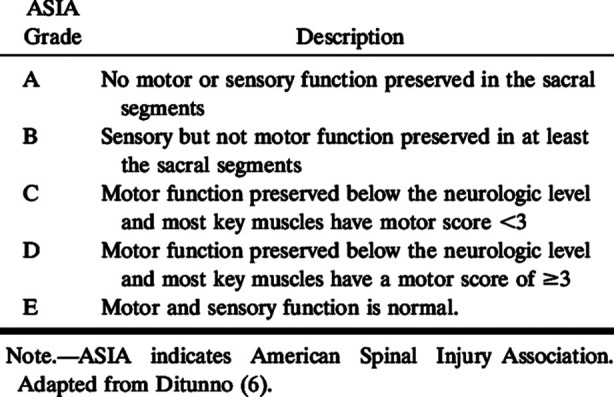
ASIA impairment scale.

## DISCUSSION

Numerous methods and procedures can be used to conduct posterior cervical fusion on patients.^12^,^13^ The LMS method is frequently used for simple screw insertion to reduce the chance of vertebral artery perforation.^14^ Our findings demonstrate that lateral mass screw fixation by Magerl technique is a reliable and effective approach for managing traumatic subaxial cervical spine bilateral facet dislocation. The mean Neck Disability Index (NDI) of 21±19.44 indicated mild disability in most patients. Neurological outcomes based on ASIA scores revealed that 86.36% of patients maintained intact neurological function postoperatively which improved from 50%, suggesting favorable clinical and functional recovery. The most commonly affected level was C5-6 (54.5%). These results underline the safety and efficacy of the procedure in stabilizing cervical spine injuries and improving functional outcomes. Jiang et al.^1^ performed a study to measure the short-term clinical outcome for Subaxial Cervical Facet Dislocations patients The results show that 52 patients with subaxial cervical facet dislocation, comprising 15 women and 37 men, were recruited in this study, either with or without neurological disability. The average age was 44.7± 29.0 (31 to 72 years old). The average pre-op NDI was 56±25 while postoperatively it improved to 19±12 after 12 weeks. Another study by Sellin et al.^4^ found that the C6-7 level accounted for the majority of injuries (37.5%, n=3). With at least three months of follow-up, the mean NDI score was 5.3 (n=6, range, 1–12; standard deviation, 4.5), which is which indicates mild impairment.

**Fig.3:**
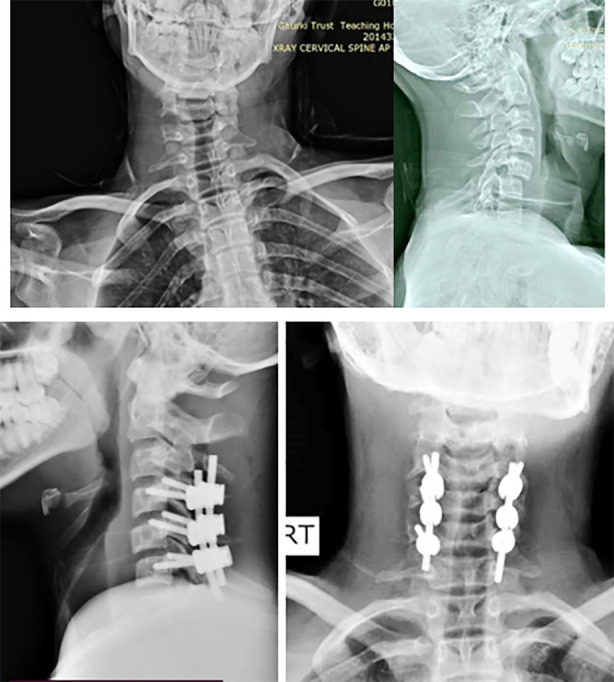
Preoperative and post operative radiology of patient with LMS fixation.

The results of this investigation confirm that LMS treatments help produce positive clinical outcomes. The patients’ functional impairment and capacity to perform everyday activities have significantly improved, as evidenced by the decline in NDI scores. Further demonstrating the reliability and stability of LMS in cervical spine procedures is the high rate of successful screw insertion and solid fusion rates. Kim et al.^15^ performed the study and the findings reveals that in the preoperative and late postoperative follow-up evaluations, there were statistically significant improvements in the mean Neck Disability Index (NDI) scores.

In our study, preoperatively 50% of the patients had intact neurology (ASIA E), followed by

ASIA D which made up of the 27.27% patients, ASIA C 9.09%, ASIA B patients (4.55%), ASIA A 9.09% illustrating the severity of the neurological dysfunction. Postoperatively no neurological recovery was observed in ASIA A or ASIA B patients, while all ASIA C and D patients showed complete recovery.These results indicate that after reduction and LMS fixation with posterior decompression, patients showed improvement in neurology. Rehman L et al^16^ conduted a study in which LMS fixation was done for facet dislocation after which there is improvement in neurology in 58% patients assessed by Frankles grade.

In our study, Fusion was accomplished in each case. No patient had the onset or worsening of a neurological impairment following surgery. Except in two cases where screws were partially pulled out, there was no evidence of implant failure at the final follow-up. Though LMS is considered safest method of screw placement in cervical spine but it has complication like lateral mass fracture, redo surgery and surgical site infection which is more common than the cervical pedicle screw placement.^17^

### Limitations:

This study has some limitations like retrospective study methodology, limited sample size, single centre and brief follow-up time. Future multicenter studies with larger sample sizes, longer follow-ups, and comparative analyses would help validate and expand upon our findings.

## CONCLUSION

Lateral mass screws are a safe and dependable method of cervical fixation that, following a satisfactory reduction, not only stabilizes the cervical spine but also promotes a patient’s great functional recovery in terms of NDI and Neurology.

### Authors’ Contribution:

**AA, SKK:** Study design, questionnaire design, data interpretation, and provided feedback through critical manuscript review. **SH, IB:** Study concept, study design, literature search, data collection, analysis, and interpretation. **LK:** Contributed to the literature search and manuscript writing. All authors have read the final version and are responsible and accountable for the accuracy and integrity of the work.
